# Trends and disparities in chronic ischemic heart disease mortality among adult cancer patients: a nationwide CDC WONDER analysis (1999–2020)

**DOI:** 10.1186/s40959-025-00409-3

**Published:** 2025-12-19

**Authors:** Soban Ali Qasim, Iftikhar khan, Syed Saad Ul Hassan, Shree Rath, Mishaim Khan, Saif Ur Rahman, Hussnain Zafar, Danish Ali Ashraf, Muhammad Abdullah Ali, Kamil Ahmad Kamil

**Affiliations:** 1 Multan Medical and Dental College, Multan, Pakistan; 2 FMH College of Medicine and dentistry Lahore, Lahore, Pakistan; 3 Department of Medicine, Allama Iqbal Medical College, Lahore, Pakistan; 4 All India Institute of Medical Sciences Bhubaneswar, Bhubaneswar, India; 5 Bacha Khan Medical College, Mardan, Pakistan; 6 University of Oklahoma Health Sciences Center, Oklahoma, USA; 7 Department of Internal Medicine, Trugift Health LLC, Wilmington, Delaware, Wilmington, USA; 8 Khyber Medical College, Peshawar, Pakistan; 9 Internal Medicine Department, Mirwais Regional Hospital, Kandahar, Afghanistan

**Keywords:** Ischemic heart disease, Cancer, Mortality disparities, CDC-WONDER, Cardio-oncology

## Abstract

**Background:**

Chronic ischemic heart disease (IHD) is a leading cause of cardiovascular-related mortality worldwide, with a growing burden among cancer patients due to shared risk factors and treatment-related Cardiotoxicity. However, nationwide trends and disparities in IHD-related mortality among cancer patients remain unexplored.

**Methodology:**

This study utilized CDC WONDER mortality data from 1999 to 2020, identifying U.S. adults (≥ 25 years) with cancer (ICD-10: C00-D48) who died from chronic IHD (ICD-10: I25) as the underlying cause. Age-adjusted mortality rates (AAMRs) and annual percent changes (APCs) were calculated and stratified by gender, age, race, geographic region, and urbanization level.

**Results:**

Between 1999 and 2020, there were 246,664 Chronic IHD-related deaths among adult cancer patients. The AAMR declined significantly from 8.44 per 100,000 in 1999 to 3.71 in 2020. A steady decline occurred from 1999 to 2018 (APC: -3.85%; 95% CI: -4.01 to -3.72), followed by a slight increase from 2018 to 2020 (APC: 2.92%; 95% CI: 0.33 to 4.70). Men had higher AAMRs than women (8.16 vs. 3.25). The highest CMR were observed in older adults (24.19), with significantly lower rates in middle-aged (1.26) and young adults (0.04). Racial disparities revealed the highest AAMRs in non-Hispanic Black individuals (5.64), followed by non-Hispanic Whites (5.37), NH American Indian (3.59), Hispanics (3.28), and NH Asians (2.7). Geographic trends showed that the Northeast had the highest AAMRs (6.88), while urban areas had slightly higher mortality than rural areas (5.23 vs. 5.06).

**Conclusions:**

This nationwide analysis highlights a significant decline in chronic IHD-related mortality among cancer patients, with persistent disparities by gender, age, race, and geographic location. The recent rise in AAMRs after 2018 suggests emerging risk factors that warrant further investigation. Addressing these disparities through targeted cardiovascular risk management in cancer patients is crucial to improving long-term outcomes.

**Supplementary Information:**

The online version contains supplementary material available at 10.1186/s40959-025-00409-3.

## Introduction

Ischemic heart disease (IHD) remains one of the most prevalent and fatal cardiovascular conditions worldwide, with an estimated burden exceeding 126 million cases and accounting for nearly 20% of all global deaths [1–3]. With improved cancer survival, cardiovascular disease has emerged as a leading cause of non-cancer mortality among cancer survivors, underscoring the importance of cardio-oncology focused population-level assessments.

The interrelationship between cancer and IHD involves multiple immunological, genetic and endocrine-mediated pathways. Certain genetic and epigenetic conditions predispose a patient to numerous synchronous malignant lesions as well as heart diseases. Some identified genes include JAK2, TTN and ATM among others. Studies predict a DNA damage repair dysfunction in contributing to combined morbidities of cancer and IHD [4]. Further, microRNAs like miR-1 and miR-133 have shown associations with both conditions. Certain chemokines released by cancer cells can cause muscle wasting and lead to insulin depletion, thus impairing the source of energy for cardiac muscles [5].

Beyond inherent disease biology, cancer therapies themselves contribute to cardiovascular risk. Agents such as doxorubicin, cisplatin, and trastuzumab commonly used in breast and gastrointestinal cancers are associated with chemotherapy-related cardiac dysfunction, including IHD and left ventricular dysfunction [6]. Radiotherapy has similarly been implicated, with a dose-dependent increase in IHD events observed in breast cancer survivors [7].

A study in the UK identified five major clusters of risk factors for IHD, which included endocrine conditions, mental disorders, respiratory diseases and cancer among others [8]. Of these, due to the exponentially rising burden of malignancies, the complex relationship between cancer and IHD warrants further investigation. A population study in Japan identified a 3.26-fold increase in mortality for IHD in those with cancer [9]. Similarly, IHD accounts for the highest cardiovascular-related mortality among cancer patients, causing almost 55% of all deaths. This was further accentuated in men and among those with older ages [10]. However, national-level analyses specifically evaluating chronic ischemic heart disease mortality among individuals with cancer remain limited, particularly in understanding demographic and geographic disparities over time.

Despite the growing recognition of the cardio-oncology interface, nationwide data on chronic IHD-related mortality among cancer patients remain limited. In this retrospective analysis of the CDC-WONDER database from 1999 to 2020, we aim to characterize temporal trends and demographic disparities in chronic IHD mortality where malignancy is listed as a contributing cause of death. By elucidating these patterns, we seek to inform clinical practice and policy aimed at mitigating cardiovascular risk among cancer populations.

## Methods

### Study setting and population

We analyzed US death certificate data from the CDC WONDER database (1999–2020) [11], using ICD-10 codes I25 (chronic ischemic heart disease) and C00–D48 (neoplasms) [12]. Cases with chronic IHD as the underlying cause and patients with neoplasms listed as contributing causes were identified from the Multiple Cause-of-Death database. Institutional review board approval was unnecessary as the study used de-identified public data and adhered to STROBE guidelines [13].

### Data abstraction

We abstracted demographic data from death certificates (1999–2020), including age, gender, race/ethnicity, urbanization, and place of death. Place of death included inpatient/outpatient facilities, emergency rooms, sudden deaths, residences, hospice/nursing homes, long-term care, or unspecified locations. Race/ethnicity was categorized as Non-Hispanic (American Indian/Alaska Native, Asian/Pacific Islander, Black/African American, White) or Hispanic/Latino. Counties were classified by population size into urban (>1 million), medium/small metropolitan (50,000–999,999), and rural (< 50,000). US regions followed Census Bureau categories (Northeast, Midwest, South, West) [14]. Age groups were young adults (25–44 years), middle-aged adults (45–64 years), and older individuals (65–85 + years). This data aligns with previous CDC WONDER studies [11, 12, 15].

### Statistical analysis

We calculated crude and Age-Adjusted Mortality Rates (AAMRs, per 100,000) with 95% confidence intervals by gender, race/ethnicity, census region, and urbanization from 1999 to 2020, using the 2000 US population as the standard. Crude Mortality Rates (CMR) were derived by dividing deaths from neoplasms and ischemic heart disease (IHD) by annual population totals [16]. We analyzed annual mortality trends using Joinpoint regression (version 5.2.0.0) [17] to estimate Annual Percent Changes (APCs) and their statistical significance, and we additionally computed the Average Annual Percent Change (AAPC) to summarize overall trends across the full study period. APCs and AAPCs significantly different from zero (two-tailed t-test; *P* < 0.05) indicated meaningful trends.

## Results

### Overall

**Fig. 1:**
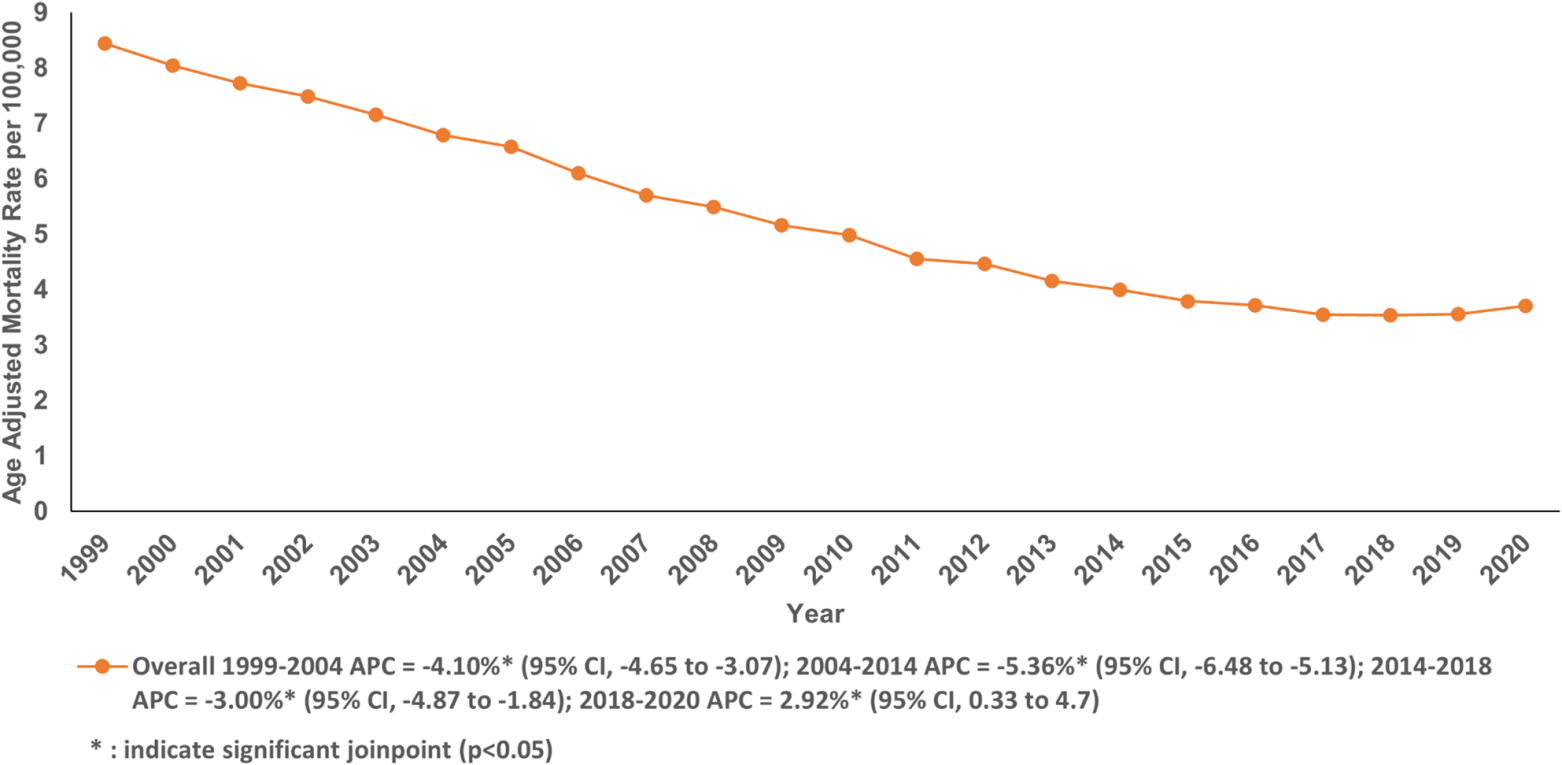
Age-adjusted mortality rate per 100,000 for Chronic Ischemic Disease in adult cancer patients

Between 1999 and 2020, a total of 246,664 cancer-related deaths (chronic IHD as the underlying cause with neoplasms listed as contributing causes) occurred, resulting in an AAMR of 5.19 per 100,000 individuals (95% CI: 5.17 to 5.21) (Table S1 and S2). The AAMR significantly decreased over the two-decade span, falling from 8.44 in 1999 to 3.71 in 2020 with an AAPC of −3.85% (95% CI: −4.01 to −3.72; *P* < 0.001). In the period from 1999 to 2004, there was a significant decline in AAMR with an APC of −4.10% (95% CI: −4.65 to −3.07; *P* < 0.001), followed by a steeper decline from 2004 to 2014 with an APC of −5.36% (95% CI: −6.48 to −5.13; *P* < 0.001) and then a relatively gradual decline from 2014 to 2018 with an APC of −3.00% (95% CI: −4.87 to −1.84; *P* < 0.001). This declining trend was interrupted by a rise in AAMR from 2018 to 2020 with an APC of 2.92% (95% CI: 0.33 to 4.70; *P* = 0.03) (Fig. 1; Table S2 and Table S3). Most deaths occurred in the decedent’s home (31.08%), followed by the medical facilities - Inpatient (27.22%), nursing home or long-term care (25.33%), and medical facility – Outpatient or ER (9.10%) (Fig. 1; Table S4).

### Demographic trends

#### Sex

**Fig. 2:**
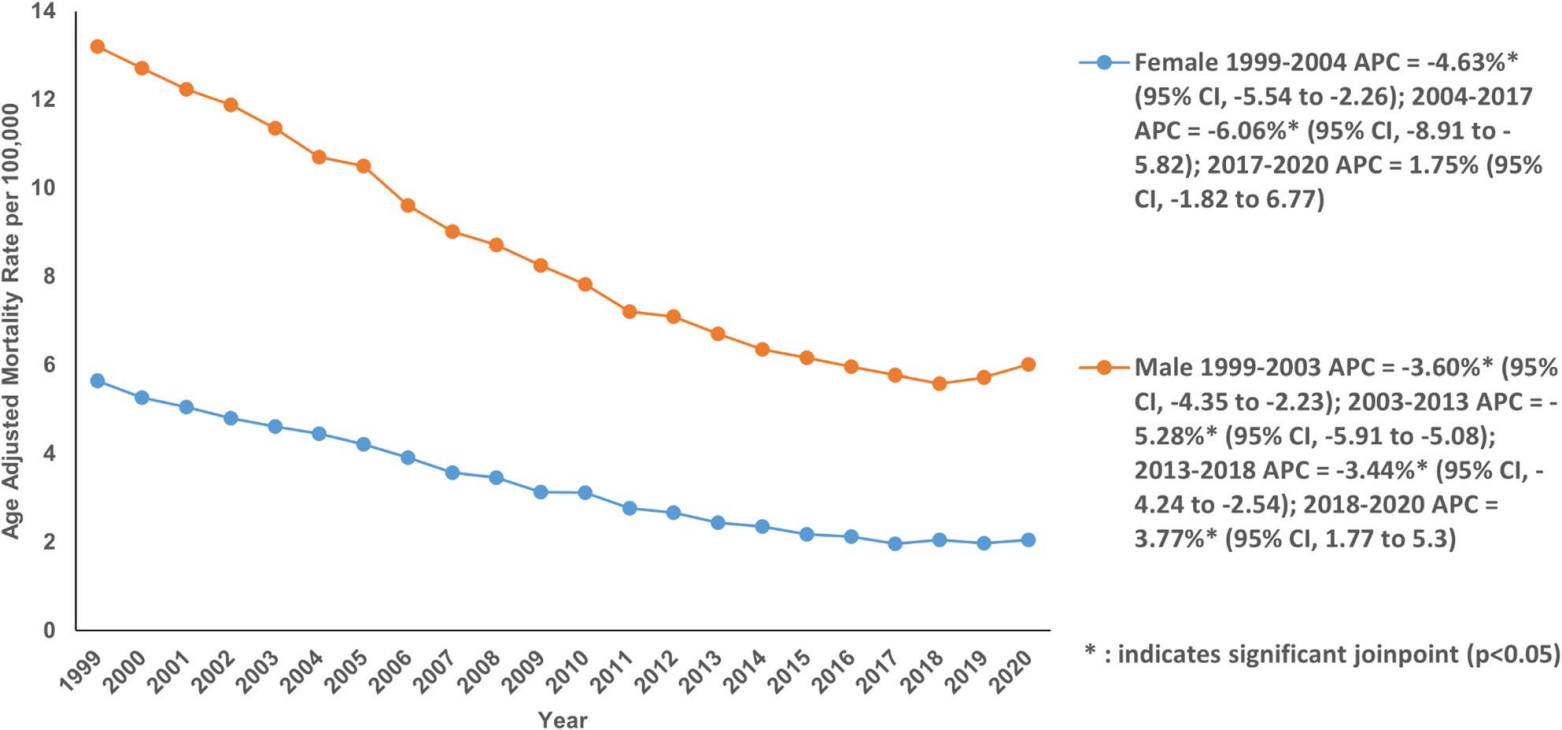
Age-adjusted mortality rate per 100,000 for Chronic Ischemic Disease in adult cancer patients, stratified by gender in the United States, 1999–2020

A higher AAMR was exhibited by males (8.16; 95% CI: 8.12 to 8.21) than females (3.25; 95% CI: 3.23 to 3.27) (Table S2). The AAMR for females decreased sharply from 5.65 in 1999 to 2.06 in 2020 with an AAPC of −4.64% (95% CI: −4.99 to −4.39; *P* < 0.001). However, their AAMR decreased in two distinct phases: a modest decline from 1999 to 2004 with an APC of −4.63% (95% CI: −5.54 to −2.26; *P* = 0.01) followed by a steep decline till 2017 with an APC of −6.06% (95% CI: −8.91 to −5.82; *P* < 0.001). From 2017 to 2020, the AAMR remained stable (Fig. 2; Table S2 and Table S3). Males also experienced a significant decrease in AAMR from 13.20 in 1999 to 6.01 in 2020, giving an AAPC of −3.69% (95% CI: −3.82 to −3.57; *P* < 0.001). The AAMR decreased in 3 segments; from 1999 to 2003 with an APC of −3.60% (95% CI: −4.35 to −2.23; *P* < 0.001), from 2003 to 2013 with an APC of −5.28% (95% CI: −5.91 to −5.08; *P* < 0.001) and from 2013 to 2018 with an APC of −3.44% (95% CI: −4.24 to −2.54; *P* < 0.001). This was followed by a significant increase from 2018 to 2020 with an APC of 3.77% (95% CI: 1.77 to 5.30; *P* < 0.001) (Fig. 2; Table S2 and Table S3).

#### Race

**Fig. 3:**
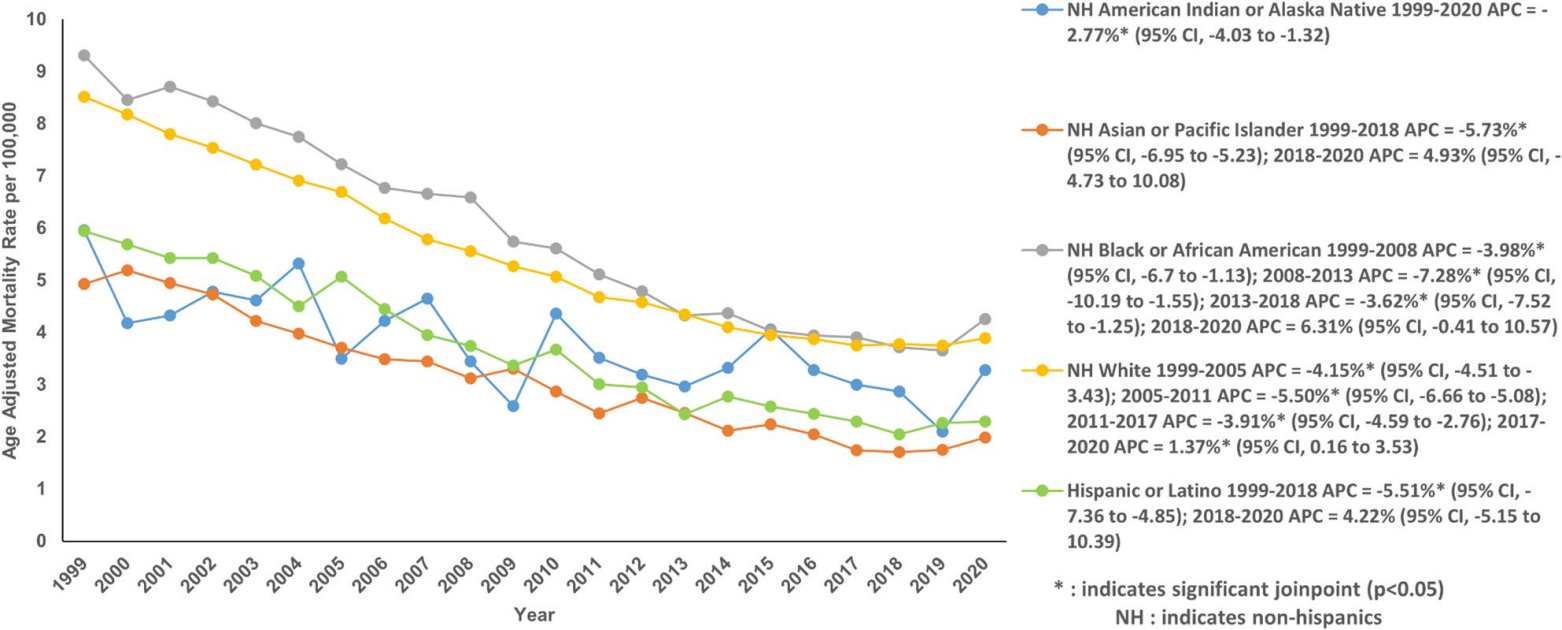
Age-adjusted mortality rate per 100,000 for Chronic Ischemic Disease in adult cancer patients, stratified by race in the United States, 1999–2020

When stratified by race/ethnicity, non-Hispanic (NH) Whites reported the highest number of deaths (207,144), however, the highest AAMR was reported in NH Blacks (5.64; 95% CI: 5.57 to 5.72) (Table S1 and Table S5). NH Blacks showed a statistically significant decline in AAMR from 9.31 in 1999 to 4.26 in 2020 with an AAPC of −3.76% (95% CI: −4.20 to −3.47; *P* < 0.001). Their AAMR decreased sharply from 1999 to 2008 with an APC of −3.98% (95% CI: −6.70 to −1.13; *P* = 0.03), followed by a much steeper decline from 2008 to 2013 with an APC of 2008 to 2013 with an APC of −7.28% (95% CI: −10.19 to −1.55; *P* = 0.004). The last segment of decreasing AAMR from 2013 to 2018 with an APC of −3.62% (95% CI: −7.52 to −1.25; *P* = 0.004) was succeeded by a period of stable AAMR till 2020 (Fig. 3; Table S3 and Table S5). NH Whites showed an overall decrease in AAMR from 8.52 in 1999 to 3.89 in 2020 with an AAPC of −3.70% (95% CI: −3.82 to −3.59; *P* < 0.001). From 1999 to 2005, there was a decline with an APC of −4.15% (95% CI: −4.51 to −3.43; *P* < 0.001). The AAMR kept on decreasing from 2005 to 2011 with an APC of −5.50% (95% CI: −6.66 to −5.08; *P* < 0.001) and then from 2011 to 2017 with an APC of −3.91% (95% CI: −4.59 to −2.76; *P* < 0.001), followed by an unusual trend of increasing AAMR till 2020 with an APC of 1.37% (95% CI: 0.16 to 3.53; *P* = 0.03) (Fig. 3; Table S3 and Table S5). The AAMR for NH American Indians decreased sharply in a single phase from 5.96 in 1999 to 3.28 in 2020 with an AAPC of −2.77% (95% CI: −4.03 to −1.32; *P* < 0.001) (Fig. 3; Table S3 and Table S5). The analysis of NH Asians and Hispanics highlighted a similar downward overall trend from 1999 to 2020 with an AAPC of −4.76% (95% CI: −5.52 to −4.32; *P* < 0.001) and AAPC of −4.63% (95% CI: −5.47 to −4.13; *P* < 0.001) respectively. The decline was mainly from 1999 to 2018 in both groups, followed by an insignificant increase in AAMR by 2020 (Fig. 3; Table S3 and Table S5).

####  Age groups

The highest CMR per 100,000 was shown by the 65 + years old age group (24.19; 95% 24.09 to 24.29). This was followed by the 45–64-year-old age group (1.26; 95% CI: 1.25 to 1.28) and 25–44-year-old age group (0.04; 95% CI: 0.03 to 0.04) respectively (Table S6).

**Fig. 4:**
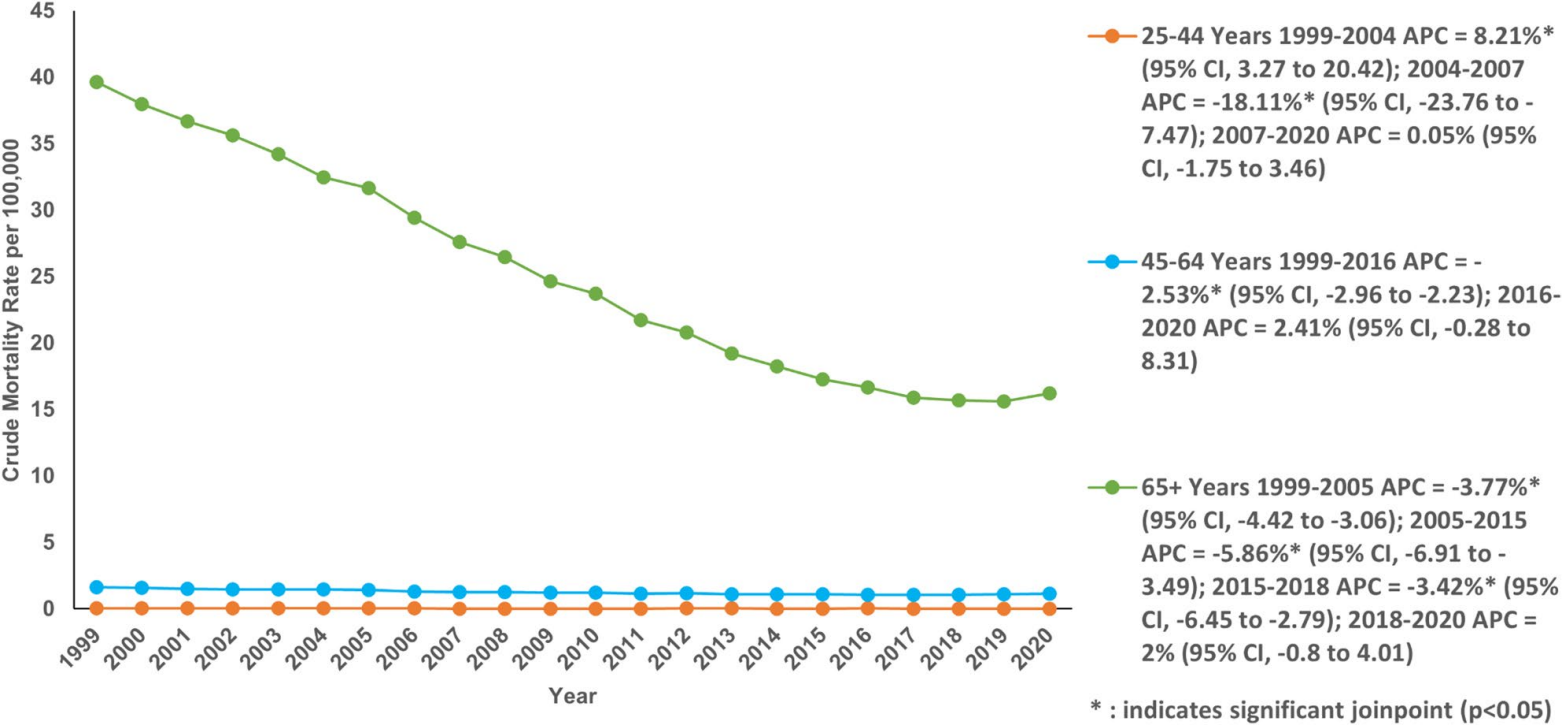
A Crude mortality rate per 100,000 for Chronic Ischemic Disease in adult cancer patients, stratified by age in the United States, 1999–2020, Older Adults (65–85 + years), Middle-Aged Adults (45–64 years). Younger Adults (25–44 years)

The sharpest decline in CMR was seen in the 65 + years old with an AAPC of −4.19% (95% CI: −4.37 to −4.07; *P* < 0.001) from 40.15 in 1999 to 17.11 in 2020. The trend was divided into 4 segments, first three segments showed a declining AAMR, from 1999 to 2005 with an APC of −3.77% (95% CI: −4.42 to −3.06; *P* < 0.001), from 2005 to 2015 with an APC of −5.86% (95% CI: −6.91 to −3.49; *P* < 0.001), from 2015 to 2018 with an APC of −3.42% (95% CI: −6.45 to −2.79; *P* < 0.001) (Fig. 4; Table S3 and Table S6). In the 45–64 years old, the CMR decreased from 1.66 in 1999 to 0.99 in 2020 with an AAPC of −1.61% (95% CI: −1.99 to −1.36; *P* < 0.001). This included a segment of decline from 1999 to 2016 with an APC of −2.53% (95% CI: −2.96 to −2.23; *P* < 0.001). There was no significant change in the CMR of 25–44 years over the course of two decades, however there was an alarming increase in the CMR from 1999 to 2004 with an APC of 8.21% (95% CI: 3.27 to 20.42; *P* < 0.001), followed by an extremely steep decline from 2004 to 2007 with an APC of −18.11% (95% CI: −23.76 to −7.47; *P* < 0.001) and then the AAMR remained stable till 2020 (Fig. 4; Table S3 and Table S6).

### Geographic patterns

#### Urban/Rural

**Fig. 5:**
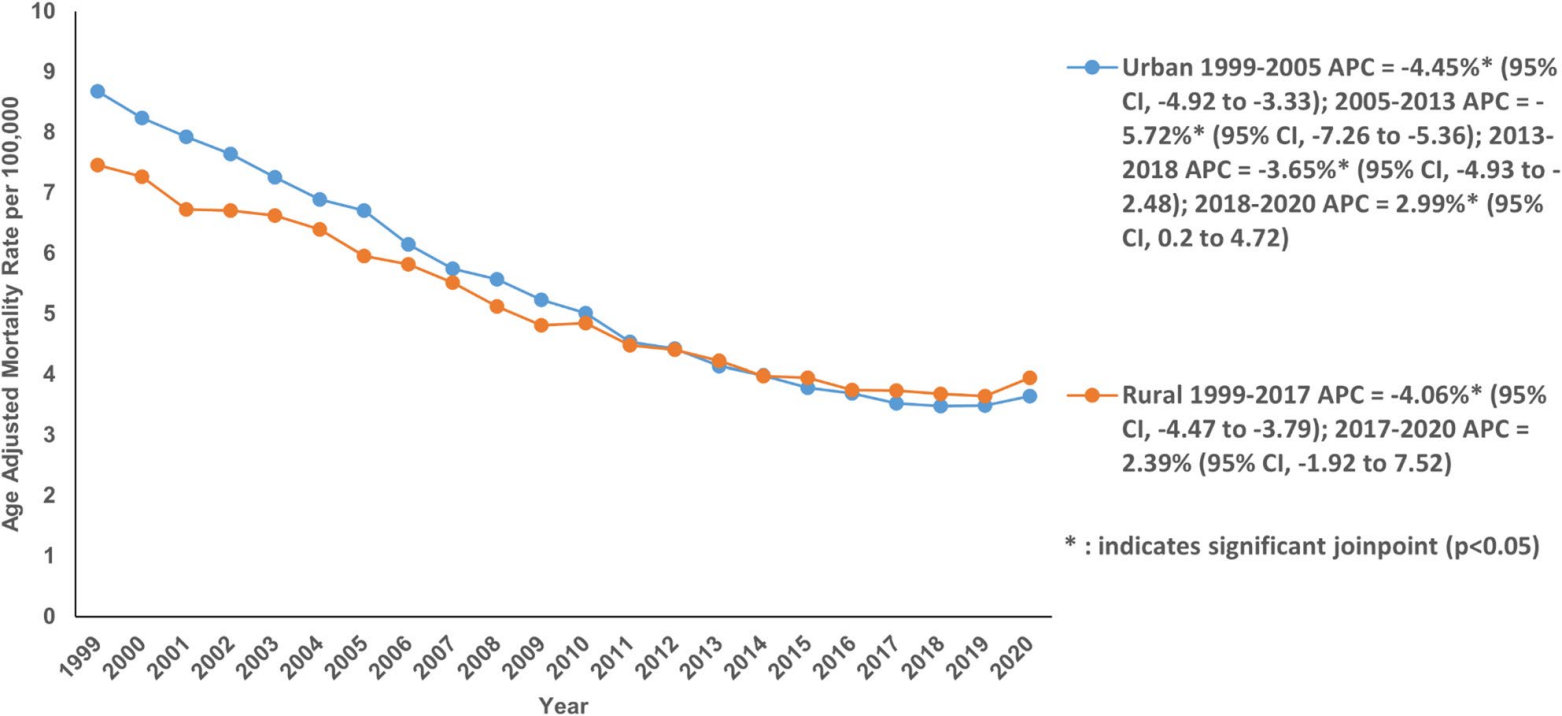
Age-adjusted mortality rate per 100,000 for Chronic Ischemic Disease in adult cancer patients in Urban and Rural areas in the United States, 1999–2020

Slightly higher AAMRs were observed in urban areas (5.23; 95% CI: 5.21 to 5.25) than in rural areas (5.06; 95% CI: 5.01 to 5.11) (Table S7). There was a significant decline in AAMRs of urban areas with an AAPC of −2.70% (95% CI: −3.09 to −2.21; *P* < 0.001) from 8.68 in 1999 to 3.65 in 2020. The analysis of individual trend segments revealed that the AAMRs decreased significantly from 1999 to 2005 with an APC of −4.45% (95% CI: −4.92 to −3.33; *P* < 0.001), from 2005 to 2013 with an APC of −5.72% (95% CI: −7.26 to −5.36; *P* < 0.001) and from 2013 to 2018 with an APC of −3.65% (95% CI: −4.93 to −2.48; *P* < 0.001). This was followed by an increasing AAMR segment from 2018 to 2020 with an APC of 2.99% (95% CI: 0.2 to 4.72; *P* = 0.03) (Fig. 5; Table S3 and Table S7). The AAMR in rural areas followed a similar trend decreasing from 7.46 in 1999 to 3.95 in 2020 with an AAPC of −3.16% (95% CI: −3.60 to −2.92; *P* < 0.001). The single phase of decline in AAMR was from 1999 to 2017 with an APC of −4.06% (95% CI: −4.47 to −3.79; *P* = 0.002) (Fig. 5; Table S3 and Table S7).

### Census region and statewide

When stratified by census region, the Northeast showed the highest AAMR (6.88; 95% CI: 6.83 to 6.94) and the highest number of fatalities was reported by the South (72,525) (Table S8).

**Fig. 6:**
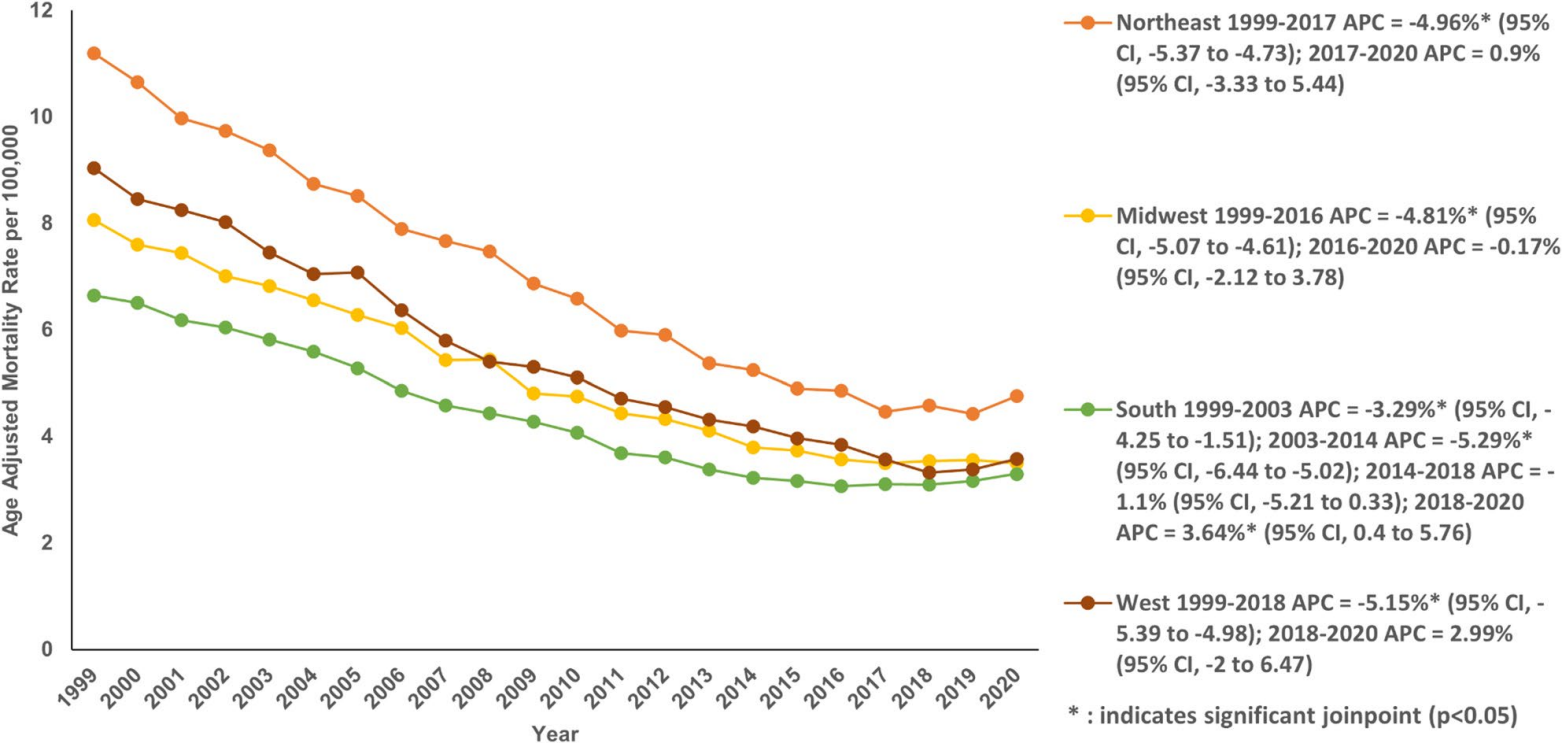
Age-adjusted mortality rate per 100,000 for Chronic Ischemic Disease in adult cancer patients, stratified by census region in the United States, 1999–2020

The AAMR in the Northeast declined from 11.2 in 1999 to 4.76 in 2020 with an AAPC of −4.14% (95% CI: −4.61 to −3.94; *P* < 0.001). In the South, AAMR decreased from 6.64 in 1999 to 3.30 in 2020 with an AAPC of −3.30% (95% CI: −3.51 to −3.13; *P* < 0.001). The West demonstrated a segment of sharp fall in AAMR from 9.04 in 1999 to 3.58 in 2020 with an AAPC of −4.40% (95% CI: −4.7 to −4.12; *P* < 0.001). The AAMR of the Midwest had a drastic decrease from 8.06 in 1999 to 3.50 in 2020 with an AAPC of −3.95% (95% CI: −4.2 to −3.74; *P* < 0.001) (Fig. 6; Table S3 and Table S8).

**Fig. 7:**
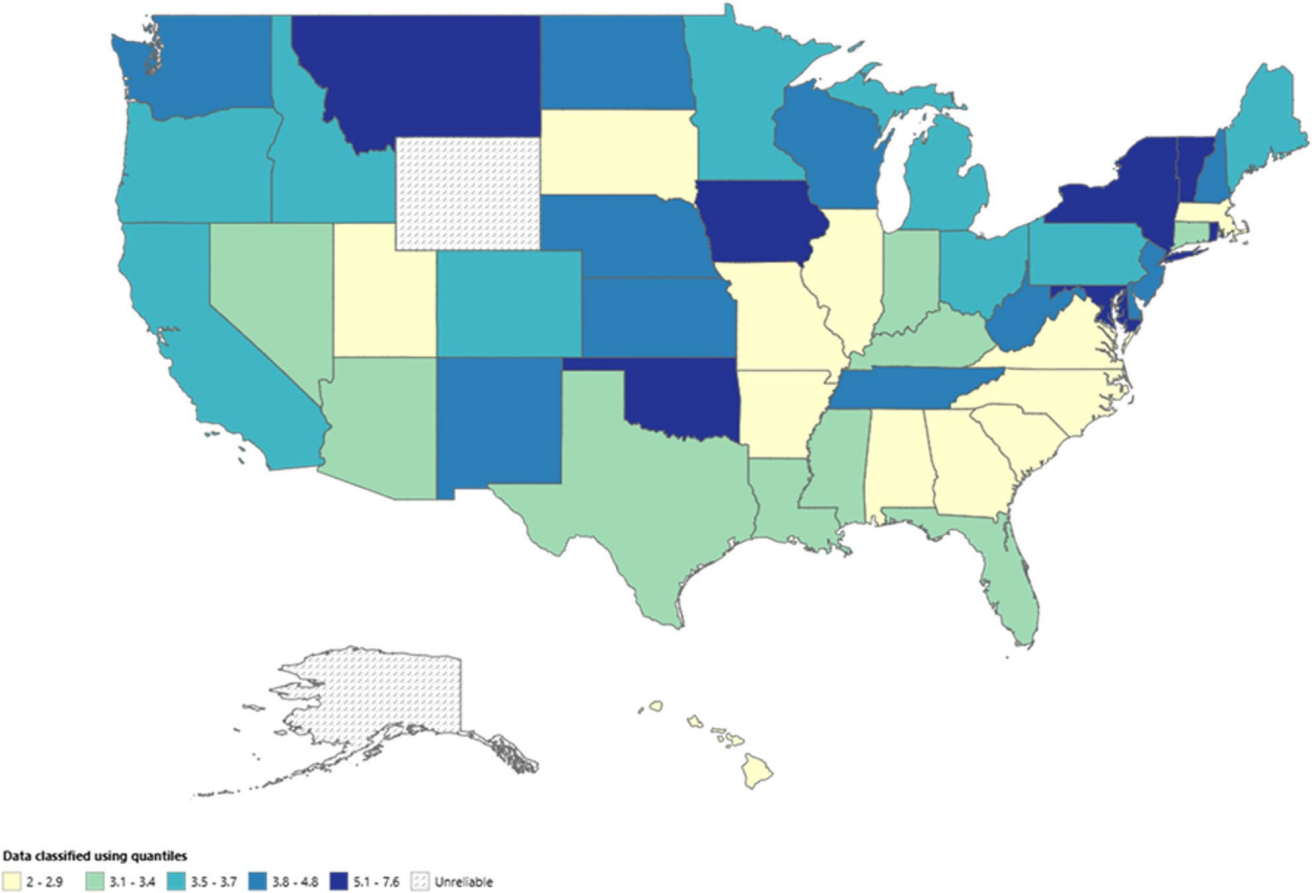
Age-adjusted mortality rate per 100,000 for Chronic Ischemic Disease in adult cancer patients, stratified by State in the United States, 1999–2020

When stratified by states, AAMR ranged from 2.34 in Utah to 9.59 in New York. The District of Columbia and the states of New York and Rhode Island were included in the top 90th percentile while the states of Utah, Georgia and Alabama were in the bottom 10th percentile (Fig. 7; Table S9).

### Cancer subtypes

**Fig. 8:**
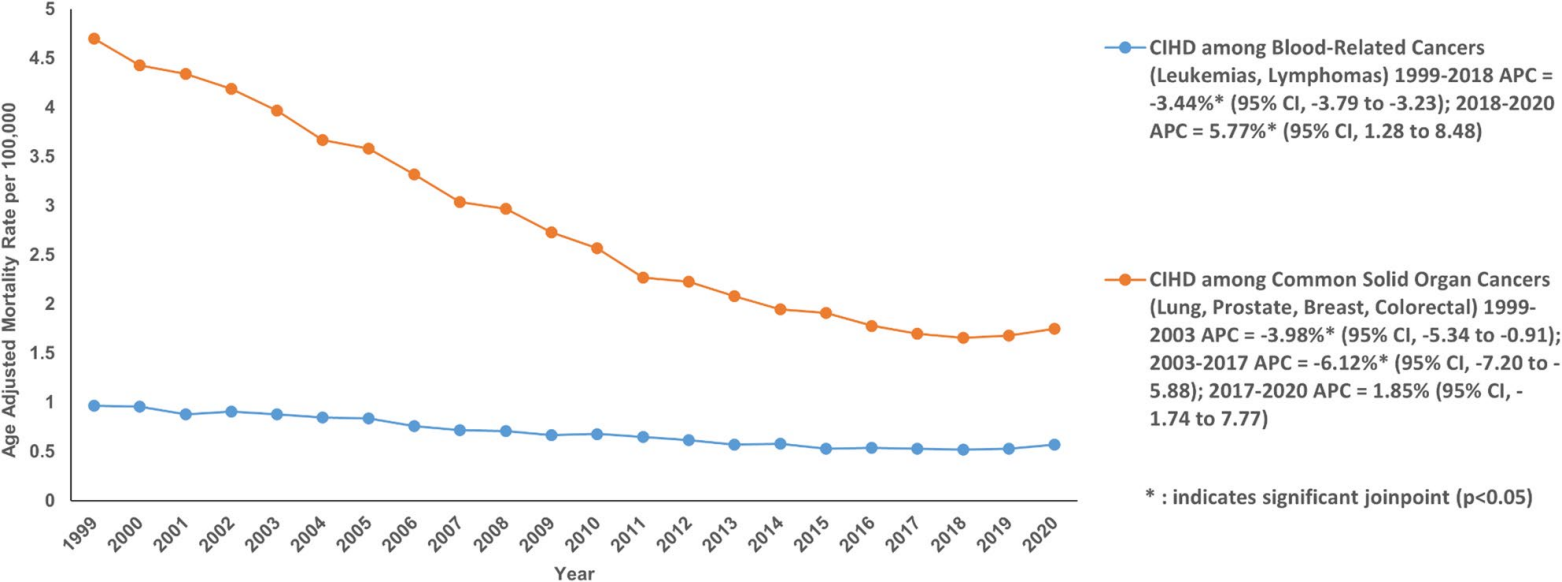
Age- Age-adjusted mortality rate per 100,000 for Chronic Ischemic Disease in adult cancer patients, stratified by Cancer subtypes

When stratified by Solid Organ Cancers (Lung, Prostate, Breast, Colorectal), the AAMR related to chronic ischemic disease among these neoplasm declined from 1999 to 2020 with an AAPC of −4.62% (95% CI: −4.97 to −4.35; *P* < 0.001). From 1999 to 2003, AAMR decreased with an APC of −3.99% (95% CI: −5.34 to −0.91; *P* = 0.02) followed by decreasing AAMR from 2003 to 2017 with an APC of −6.13% (95% CI: −7.20 to −5.88; *P* < 0.001). The AAMR increased from 2017 to 2020 but the increase was not statistically significant. The AAMR related to chronic ischemic disease among hematological cancers (Lymphoid leukemia, Myeloid leukemia, Monocytic leukemia, Hodgkin lymphoma, Non-Hodgkin lymphoma, etc.) had decreasing trend from 1999 to 2020 with an AAPC of −2.60% (95% CI: −2.88 to −2.40; *P* < 0.001). With an APC of −3.44% (95% CI: −3.79 to −3.23; *P* < 0.001), AAMR decreased from 1999 to 2018. This was followed by a sharp increase in AAMR from 2018 to 2020 with an APC of 5.77% (95% CI: 1.29 to 8.49; *P* = 0.01) (Fig. 8; Supplemental Table 10).

A detailed breakdown of trends by cancer type including major solid organ cancers and hematologic malignancies is provided in Supplementary Tables S11 and S12.

## Discussion

This study explored the mortality trends associated with chronic ischemic heart disease (CIHD) in the presence of neoplasms as a contributing cause of death in the United States from 1999 to 2020. The findings revealed a significant decline in AAMR over the two-decade period, with notable variations across demographic groups, geographic regions, and urbanization levels. Males exhibited higher mortality rates compared to females, and non-Hispanic Blacks had the highest AAMR among racial/ethnic groups. The 65 + age group showed the highest crude mortality rates, with a sharp decline over time. Urban areas had slightly higher AAMRs than rural areas, and the Northeast region reported the highest mortality rates. The study also highlighted that most deaths occurred at home, followed by inpatient facilities and nursing homes. These findings highlight the complex interplay between IHD and neoplasms, as well as the influence of demographic and geographic factors on mortality trends.

The findings of this study align with and expand upon a wide range of existing literature on the intersection of cardiovascular disease and cancer. The observed decline in mortality rates is consistent with broader trends in cardiovascular disease mortality, as reported in previous CDC WONDER database analyses and epidemiological studies. This decline may be attributed to advancements in primary prevention, improved treatment strategies, and widespread lifestyle modifications.

However, gaps remain in understanding the differential impact of these factors across demographic groups, as well as the long-term effects of emerging cardiovascular therapies and evolving cancer treatments on patient outcomes. For instance, the study by Das et al. emphasized the shared risk factors and biological pathways between coronary artery disease (CAD) and cancer, which may explain the higher mortality rates observed in certain demographic groups [18]. Similarly, Liu et al. found that nearly one-sixth of deaths among CAD patients were due to cancer, with lung, liver, and colorectal cancers being the most common, further supporting the findings of this study [19].The higher mortality rates among males and non-Hispanic Blacks are also in line with studies highlighting disparities in cardiovascular outcomes based on sex and race. Targeted public health interventions, such as community-based screening programs, improved access to preventive care, culturally tailored health education, and initiatives to address social determinants of health, could help reduce these disparities [20]. For example, Ameri et al. discussed the growing number of cancer patients with IHD, emphasizing the role of shared risk factors such as hypercholesterolemia, obesity, and systemic inflammation [21]. This aligns with the findings of this study, which showed higher mortality rates among males and non-Hispanic Blacks. Additionally, Lee et al. reported that smoking, a major risk factor for both IHD and cancer, is associated with higher mortality rates, particularly in males, which may explain the observed disparities [22].

The role of urbanization and geographic disparities in mortality rates is also supported by previous research. Jiang et al. highlighted the impact of environmental factors, such as Helicobacter pylori infection, on both gastric cancer and cardiovascular disease, which may contribute to the observed differences in mortality rates between urban and rural areas [23]. Similarly, Pal et al. demonstrated the impact of environmental interventions, such as reducing arsenic levels in drinking water, on reducing mortality from ischemic heart disease and cancer, suggesting that geographic and environmental factors play a significant role in shaping mortality trends [24].

The findings of this study also resonate with the growing body of literature on the cardio-oncology field, which examines the complex relationship between cancer and cardiovascular disease. For example, Nochioka et al. emphasized the increased risk of stroke and systemic thrombosis in patients with a history of cancer and atrial fibrillation, suggesting that the interplay between cancer and cardiovascular disease may exacerbate mortality risks [25]. This aligns with the observed increase in mortality rates from 2018 to 2020, which may be attributed to the growing prevalence of cancer survivors with cardiovascular complications. Furthermore, Friedlander et al. discussed the long-term cardiovascular effects of radiation therapy in cancer patients, which may contribute to the observed mortality trends [26]. Beyond the immediate topic, this study contributes to the broader discourse on the impact of lifestyle and environmental factors on chronic disease mortality. For instance, Wang et al. highlighted the role of dietary factors, such as low fibre intake, in contributing to the burden of ischemic heart disease and colorectal cancer, which may have implications for the observed mortality trends [27]. Similarly, Fraser et al. demonstrated the protective effects of a plant-based diet on cardiovascular disease and cancer mortality, suggesting that dietary interventions could play a role in reducing the burden of these diseases [28]. The study by Thompson et al. further supports the importance of diet in chronic disease prevention, showing that healthful plant-based diets are associated with lower risks of mortality, cardiovascular disease, and cancer [29]. This aligns with the findings of this study, which showed a decline in mortality rates over time, potentially reflecting improvements in dietary patterns and lifestyle interventions. The findings of this study also align with research on the impact of environmental tobacco smoke (ETS) on chronic diseases. Rios et al.reported that ETS exposure is associated with increased risks of lung cancer and ischemic heart disease, which may contribute to the observed mortality trends in this study [30]. Similarly, Tas et al. highlighted the impact of comorbidities such as hypertension, diabetes, and chronic obstructive pulmonary disease (COPD) on cancer outcomes, suggesting that the presence of multiple comorbidities may exacerbate mortality risks [31]. The study by Garduno et al. discussed the connection between heart failure and cancer, emphasizing the role of chronic activation of the renin-angiotensin-aldosterone system in both conditions [32]. This aligns with the findings of this study, which showed a significant burden of mortality from IHD and neoplasms, particularly among older adults.

Additionally, Qi et al. explored the role of autophagy in cancer and myocardial ischemia-reperfusion injury, suggesting that targeting autophagy pathways could provide new therapeutic strategies for these diseases [33]. The findings of this study also resonate with research on the impact of radiation exposure on chronic diseases. Boice Jr. reported that prolonged exposure to low-dose radiation increased the risk of leukaemia and other cancers, which may contribute to the observed mortality trends in this study [34]. Similarly, Friedlander et al. discussed the long-term cardiovascular effects of radiation therapy in cancer patients, suggesting that radiation-induced heart disease may contribute to the burden of IHD mortality. Finally, the study by Media et al. highlighted the impact of comorbidities such as COPD on non-small cell lung cancer (NSCLC) outcomes, suggesting that the presence of multiple comorbidities may exacerbate mortality risks [35]. This aligns with the findings of this study, which showed a significant burden of mortality from IHD and neoplasms, particularly among older adults.

It is important to contextualize our findings against general population trends. Studies in the broader U.S. population, including Raisi-Estabragh et al., Wolf et al., and more recently Kwaah et al., demonstrate a parallel decline in CIHD mortality during the same period [2, 10, 36], specifically highlighted persistent disparities across sex, race, and urbanization, with males and non-Hispanic Black individuals experiencing disproportionately higher mortality despite overall improvements. Similarly, our results suggest that while overall declines are consistent, disparities remain more pronounced among cancer patients, particularly in males, older adults, and non-Hispanic Black populations. This indicates that observed trends are not merely reflective of general population improvements but highlight a disproportionate cardiovascular burden uniquely affecting cancer survivors.

In addition to general population comparisons, it is also informative to contrast chronic IHD mortality with acute myocardial infarction (AMI) trends among cancer patients. Saeed et al. (2025) reported that AMI-related mortality in this population declined markedly between 1999 and 2015 but subsequently stabilized through 2020, with disparities persisting by sex, race, age, and urbanization [37]. By comparison, our analysis of chronic IHD demonstrates a continued, albeit slower, decline through 2020. This divergence may reflect differences in therapeutic advances, as acute cardiac care has benefitted from rapid improvements in reperfusion strategies, emergency response systems, and evidence-based secondary prevention [38, 39]. In contrast, chronic IHD management relies more heavily on long-term lifestyle modification, adherence to preventive therapies, and ongoing access to care, factors that may be more vulnerable to social and structural disparities [40, 41]. Together, these findings underscore that while progress has been made in reducing AMI-related mortality, chronic IHD continues to exert a disproportionate burden on cancer patients, highlighting the importance of sustained, multidisciplinary cardio-oncology strategies that address both acute and chronic disease pathways.

The findings of this study carry several implications for clinical practice and public health. First, the persistence of disparities by race, sex, and geography underscores the need for targeted cardio-oncology interventions, particularly in high-risk subgroups. Second, the recent uptick in IHD mortality after 2018 highlights the importance of continued surveillance and early detection of cardiovascular complications in cancer survivors. Integration of routine cardiovascular risk screening into oncology care, adoption of multidisciplinary cardio-oncology clinics, and incorporation of preventive strategies such as lifestyle counseling and pharmacologic cardioprotection may help reduce long-term mortality. Future studies that combine population-level data with granular clinical and behavioral information will be critical to developing precision approaches that address both biological and social determinants of health.

This study has notable strengths, including the use of a large, nationally representative CDC WONDER dataset covering a 22-year period, allowing robust analysis of mortality trends. By examining demographic and geographic subgroups, using AAMRs, and applying Joinpoint regression analysis, significant trends and shifts over time were effectively identified. However, several limitations exist. First, reliance on death certificate data may introduce coding errors or misclassification, particularly when multiple comorbidities, such as IHD and neoplasms, are involved. Second, individual-level risk factors, including smoking, obesity, and physical activity, were not accounted for, a common limitation of administrative data-based studies. Third, the analysis focused solely on mortality rates without exploring the underlying mechanisms driving observed disparities. Finally, the use of aggregate mortality data from the CDC WONDER database did not allow for multivariate adjustment for potential confounders such as socioeconomic status, comorbidities, or treatment differences. As a result, causal inferences cannot be drawn, and residual confounding may partially explain the observed disparities [42]. Despite these limitations, the study fills important gaps by detailing mortality trends related to IHD and neoplasms, highlighting disparities among high-risk populations such as males, non-Hispanic Blacks, and older adults. It also emphasizes the significance of geographic and environmental factors, which can inform targeted interventions, public health policy, and resource allocation. Future research incorporating clinical and behavioural data could further elucidate factors contributing to these trends.

This study provides insights into U.S. mortality trends for CIHD with neoplasms as contributing causes (1999–2020). Findings indicate significant declines in mortality, though notable disparities remain across demographic and geographic subgroups. Addressing these disparities requires community-based screening, equitable preventive care, culturally tailored education, and interventions targeting modifiable risk factors such as smoking, diet, and physical activity. Tackling social determinants, including healthcare access, environmental factors, and economic inequalities, is crucial. Future research should explore underlying mechanisms and individual-level risk factors. A multidisciplinary approach focused on reducing disparities could further improve outcomes for high-risk populations.

## Supplementary Information

Supplementary Material 1.

## Data Availability

The data supporting the findings of this study are openly available in CDC Wonder at [https://wonder.cdc.gov/].
