# Supplementary material for: Trends and disparities in chronic ischemic heart disease mortality among adult cancer patients: a nationwide CDC WONDER analysis (1999–2020)
Source: Cardiooncology. 2025 Dec 19;11:114. doi: 10.1186/s40959-025-00409-3 (PMC12717754; doi:10.1186/s40959-025-00409-3)
Supplement: Supplementary file 1 — Supplementary Material 1. [file 40959_2025_409_MOESM1_ESM.docx]

Contents

[Supplemental Table 1: 2](#_Toc198746902)

[Supplemental Table 2: 3](#_Toc198746903)

[Supplemental Table 3: 5](#_Toc198746904)

[Supplemental Table 4: 6](#_Toc198746905)

[Supplemental Table 5: 7](#_Toc198746906)

[Supplemental Table 6: 9](#_Toc198746907)

[Supplemental Table 7: 10](#_Toc198746908)

[Supplemental Table 8: 11](#_Toc198746909)

# Supplemental Table 1:

Chronic Ischemic Heart Disease Among Neoplasms-related Deaths, Stratified by Sex and Race, in the United States, 1999 to 2020

| **Year** | **Deaths** | | | | | | | |  |
| --- | --- | --- | --- | --- | --- | --- | --- | --- | --- |
|  |  |  |  |  |  |  |  |  |  |
|  | **Overall** | **Female** | **Male** | **NH White** | **NH Black or African American** | **NH Asian or Pacific Islander** | **NH American Indian or Alaska Native** | **Hispanic or Latino** |  |
| **1999** | **14834** | **6286** | **8548** | **12815** | **1314** | **169** | **37** | **436** |  |
| **2000** | **14333** | **5938** | **8395** | **12418** | **1218** | **191** | **30** | **442** |  |
| **2001** | **13957** | **5746** | **8211** | **11972** | **1259** | **190** | **30** | **454** |  |
| **2002** | **13694** | **5553** | **8141** | **11676** | **1238** | **203** | **34** | **479** |  |
| **2003** | **13328** | **5375** | **7953** | **11368** | **1204** | **192** | **32** | **481** |  |
| **2004** | **12844** | **5208** | **7636** | **10945** | **1176** | **189** | **41** | **453** |  |
| **2005** | **12687** | **5012** | **7675** | **10798** | **1126** | **195** | **29** | **518** |  |
| **2006** | **11966** | **4743** | **7223** | **10145** | **1078** | **195** | **39** | **485** |  |
| **2007** | **11440** | **4469** | **6971** | **9632** | **1095** | **199** | **38** | **461** |  |
| **2008** | **11292** | **4353** | **6939** | **9458** | **1116** | **194** | **31** | **467** |  |
| **2009** | **10784** | **4015** | **6769** | **9063** | **1019** | **220** | **24** | **437** |  |
| **2010** | **10605** | **4062** | **6543** | **8855** | **1004** | **196** | **38** | **491** |  |
| **2011** | **9984** | **3725** | **6259** | **8346** | **943** | **186** | **37** | **447** |  |
| **2012** | **9990** | **3624** | **6366** | **8313** | **928** | **223** | **35** | **458** |  |
| **2013** | **9563** | **3360** | **6203** | **8010** | **872** | **212** | **37** | **402** |  |
| **2014** | **9392** | **3343** | **6049** | **7716** | **908** | **201** | **39** | **492** |  |
| **2015** | **9213** | **3143** | **6070** | **7531** | **876** | **221** | **53** | **478** |  |
| **2016** | **9142** | **3126** | **6016** | **7477** | **878** | **221** | **46** | **479** |  |
| **2017** | **9023** | **2961** | **6062** | **7344** | **923** | **198** | **40** | **479** |  |
| **2018** | **9159** | **3128** | **6031** | **7544** | **896** | **203** | **41** | **443** |  |
| **2019** | **9401** | **3037** | **6364** | **7685** | **909** | **225** | **35** | **521** |  |
| **2020** | **10033** | **3208** | **6825** | **8033** | **1087** | **270** | **53** | **551** |  |

**NH= non-Hispanic**

# Supplemental Table 3:

Annual Percent Change (APC) and Average Annual Percent Change (AAPC) of Chronic Ischemic Heart Disease Among Neoplasms-related Age-Adjusted Rates per 100,000 in the United States, 1999 to 2020

| **Year Interval** | **APC (95% CI)** | | **Year Interval** | | **AAPC (95% CI)** | |
| --- | --- | --- | --- | --- | --- | --- |
| **Overall** | | | | | | |
| **1999-2004** | **-4.09(-4.65 to -3.07)** | | **1999-2020** | | **-3.84*(-4.01 to -3.72)** | |
| **2004-2014** | **-5.35(-6.48 to -5.13)** | |  |  |  |  |
| **2014-2018** | **-2.99(-4.87 to -1.84)** | |  |  |  |  |
| **2018-2020** | **2.92*(0.33 to 4.70)** | |  |  |  |  |
| **Female** | | | | | | |
| **1999-2004** | **-4.63(-5.54 to -2.26)** | | **1999-2020** | | **-4.64(-4.99 to -4.39)** | |
| **2004-2017** | **-6.06(-8.91 to -5.82)** | |  |  |  |  |
| **2017-2020** | **1.74(-1.82 to 6.77)** | |  |  |  |  |
| **Male** | | | | | | |
| **1999-2003** | **-3.60(-4.35 to -2.23)** | | **1999-2020** | | **-3.69(-3.82 to -3.57)** | |
| **2003-2013** | **-5.28(-5.91 to -5.08)** | |  |  |  |  |
| **2013-2018** | **-3.43(-4.24 to -2.54)** | |  |  |  |  |
| **2018-2020** | **3.77*(1.77 to 5.30)** | |  |  |  |  |
| **NH American Indian or Alaska Native** | | | | | | |
| **1999-2020** | **-2.77(-4.03 to -1.32)** | | **1999-2020** | | **-2.77(-4.03 to -1.32)** | |
| **NH Asian or Pacific Islander** | | | | | | |
| **1999-2018** | **-5.73(-6.95 to -5.23)** | | **1999-2020** | | **-4.76(-5.52 to -4.32)** | |
| **2018-2020** | **4.93(-4.73 to 10.08)** | |  |  |  |  |
| **NH Black or African American** | | | | | | |
| **1999-2008** | **-3.98(-6.70 to -1.13)** | | **1999-2020** | | **-3.76(-4.20 to -3.47)** | |
| **2008-2013** | **-7.27(-10.19 to -1.55)** | |  |  |  |  |
| **2013-2018** | **-3.62(-7.52 to -1.25)** | |  |  |  |  |
| **2018-2020** | **6.30(-0.41 to 10.57)** | |  |  |  |  |
| **NH White** | | | | | | |
| **1999-2005** | **-4.15(-4.51 to -3.43)** | | **1999-2020** | | **-3.70(-3.82 to -3.59)** | |
| **2005-2011** | **-5.50(-6.66 to -5.08)** | |  |  |  |  |
| **2011-2017** | **-3.90(-4.59 to -2.76)** | |  |  |  |  |
| **2017-2020** | **1.36*(0.16 to 3.53)** | |  |  |  |  |
| **Hispanic or Latino** | | | | | | |
| **1999-2018** | **-5.51(-7.36 to -4.85)** | | **1999-2020** | | **-4.62(-5.47 to -4.13)** | |
| **2018-2020** | **4.22(-5.15 to 10.39)** | |  |  |  |  |
| **Northeast** | | | | | | |
| **1999-2017** | **-4.95(-5.37 to -4.73)** | | **1999-2020** | | **-4.14(-4.61 to -3.94)** | |
| **2017-2020** | **0.89(-3.33 to 5.44)** | |  |  |  |  |
| **Midwest** | | | | | | |
| **1999-2016** | **-4.81(-5.07 to -4.61)** | | **1999-2020** | | **-3.94(-4.20 to -3.74)** | |
| **2016-2020** | **-0.16(-2.12 to 3.78)** | |  |  |  |  |
| **South** | | | | | | |
| **1999-2003** | **-3.29(-4.25 to -1.51)** | | **1999-2020** | | **-3.29(-3.51 to -3.13)** | |
| **2003-2014** | **-5.28(-6.44 to -5.02)** | |  |  |  |  |
| **2014-2018** | **-1.09(-5.21 to 0.33)** | |  |  |  |  |
| **2018-2020** | **3.63*(0.40 to 5.76)** | |  |  |  |  |
| **West** | | | | | | |
| **1999-2018** | **-5.15(-5.39 to -4.98)** | | **1999-2020** | | **-4.40(-4.70 to -4.12)** | |
| **2018-2020** | **2.98(-2.00 to 6.47)** | |  |  |  |  |
| **Urban or Metropolitan** | | | | | | |
| **1999-2005** | **-4.45(-4.92 to -3.33)** | | **1999-2020** | | **-4.06(-4.25 to -3.92)** | |
| **2005-2013** | **-5.71(-7.26 to -5.36)** | |  |  |  |  |
| **2013-2018** | **-3.64(-4.93 to -2.48)** | |  |  |  |  |
| **2018-2020** | **2.98*(0.20 to 4.72)** | |  |  |  |  |
| **Rural or Non-Metropolitan** | | | | | | |
| **1999-2017** | **-4.05(-4.47 to -3.79)** | | **1999-2020** | | **-3.16(-3.60 to -2.92)** | |
| **2017-2020** | **2.39(-1.92 to 7.52)** | |  |  |  |  |
| **Hematological Cancers** | | | | | | |
| **1999-2018** | | **-3.44(-3.79 to -3.23)** | | **1999-2020** | | **-2.59(-2.88 to -2.40)** |
| **2018-2020** | | **5.77*(1.29 to 8.49)** | |  |  |  |
| **Common Solid Organ Cancers** | | | | | | |
| **1999-2003** | | **-3.98(-5.34 to -0.91)** | | **1999-2020** | | **-4.61(-4.97 to -4.35)** |
| **2003-2017** | | **-6.12(-7.20 to -5.89)** | |  |  |  |
| **2017-2020** | | **1.85(-1.75 to 7.77)** | |  |  |  |

**APC= Annual Percent Changes AAPC= Average Annual Percent Changes**

*= **significant, p < 0.05**

# Supplemental Table 2:

Overall and Sex‐Stratified Chronic Ischemic Heart Disease Among Neoplasms-Related Age-Adjusted Mortality Rates per 100,000 in the United States, 1999 to 2020

| **Year** | **Age-Adjusted Rate (95% CI)** | | |
| --- | --- | --- | --- |
|  | **Male** | **Female** | **Overall** |
| **1999** | **13.2(12.92 - 13.49)** | **5.65(5.51 - 5.79)** | **8.44(8.31 - 8.58)** |
| **2000** | **12.71(12.44 - 12.99)** | **5.27(5.13 - 5.4)** | **8.04(7.91 - 8.17)** |
| **2001** | **12.24(11.97 - 12.51)** | **5.06(4.93 - 5.19)** | **7.72(7.59 - 7.84)** |
| **2002** | **11.88(11.62 - 12.15)** | **4.81(4.68 - 4.93)** | **7.48(7.35 - 7.6)** |
| **2003** | **11.36(11.1 - 11.61)** | **4.62(4.5 - 4.75)** | **7.15(7.02 - 7.27)** |
| **2004** | **10.7(10.46 - 10.95)** | **4.45(4.33 - 4.57)** | **6.79(6.67 - 6.91)** |
| **2005** | **10.5(10.27 - 10.74)** | **4.22(4.1 - 4.33)** | **6.58(6.46 - 6.69)** |
| **2006** | **9.61(9.38 - 9.83)** | **3.91(3.8 - 4.02)** | **6.1(5.99 - 6.2)** |
| **2007** | **9.02(8.8 - 9.23)** | **3.58(3.47 - 3.68)** | **5.7(5.6 - 5.81)** |
| **2008** | **8.72(8.52 - 8.93)** | **3.46(3.36 - 3.56)** | **5.49(5.39 - 5.6)** |
| **2009** | **8.25(8.05 - 8.45)** | **3.13(3.04 - 3.23)** | **5.16(5.06 - 5.26)** |
| **2010** | **7.82(7.63 - 8.01)** | **3.12(3.03 - 3.22)** | **4.98(4.88 - 5.07)** |
| **2011** | **7.21(7.03 - 7.39)** | **2.77(2.68 - 2.86)** | **4.55(4.46 - 4.64)** |
| **2012** | **7.1(6.93 - 7.28)** | **2.67(2.58 - 2.76)** | **4.46(4.37 - 4.55)** |
| **2013** | **6.7(6.54 - 6.87)** | **2.45(2.36 - 2.53)** | **4.15(4.07 - 4.23)** |
| **2014** | **6.35(6.19 - 6.51)** | **2.36(2.28 - 2.44)** | **3.99(3.9 - 4.07)** |
| **2015** | **6.16(6 - 6.31)** | **2.18(2.1 - 2.25)** | **3.79(3.71 - 3.87)** |
| **2016** | **5.96(5.81 - 6.12)** | **2.13(2.05 - 2.2)** | **3.72(3.64 - 3.8)** |
| **2017** | **5.77(5.63 - 5.92)** | **1.97(1.89 - 2.04)** | **3.55(3.48 - 3.62)** |
| **2018** | **5.59(5.44 - 5.73)** | **2.06(1.98 - 2.13)** | **3.54(3.47 - 3.62)** |
| **2019** | **5.72(5.58 - 5.87)** | **1.98(1.91 - 2.05)** | **3.56(3.48 - 3.63)** |
| **2020** | **6.01(5.86 - 6.15)** | **2.06(1.98 - 2.13)** | **3.71(3.64 - 3.78)** |
| **Average Age-Adjusted Rate** | **8.57(8.37 - 8.77)** | **3.36(3.26 - 3.46)** | **5.39(5.29 - 5.49)** |

# Supplemental Table 5:

Chronic Ischemic Heart Disease Among Neoplasms-Related Age-Adjusted Mortality Rates per 100,000 Stratified by Race in the United States, 1999 to 2020

| **Year** | **Age-Adjusted Rate (95% CI)** | | | | |
| --- | --- | --- | --- | --- | --- |
|  | **NH American Indian or Alaska Native** | **NH Asian or Pacific Islander** | **NH Black or African American** | **NH White** | **Hispanic or Latino** |
| **1999** | **5.96(4.13 - 8.33)** | **4.93(4.16 - 5.7)** | **9.31(8.8 - 9.81)** | **8.52(8.38 - 8.67)** | **5.94(5.37 - 6.51)** |
| **2000** | **4.18(2.78 - 6.04)** | **5.19(4.43 - 5.95)** | **8.46(7.98 - 8.94)** | **8.18(8.03 - 8.32)** | **5.69(5.14 - 6.23)** |
| **2001** | **4.33(2.88 - 6.26)** | **4.95(4.23 - 5.67)** | **8.71(8.23 - 9.2)** | **7.8(7.66 - 7.94)** | **5.43(4.92 - 5.95)** |
| **2002** | **4.78(3.25 - 6.78)** | **4.73(4.06 - 5.41)** | **8.43(7.95 - 8.9)** | **7.54(7.4 - 7.67)** | **5.43(4.93 - 5.94)** |
| **2003** | **4.62(3.12 - 6.6)** | **4.22(3.6 - 4.83)** | **8.01(7.55 - 8.47)** | **7.22(7.09 - 7.35)** | **5.09(4.62 - 5.56)** |
| **2004** | **5.32(3.74 - 7.33)** | **3.98(3.4 - 4.56)** | **7.75(7.3 - 8.2)** | **6.91(6.78 - 7.04)** | **4.5(4.08 - 4.93)** |
| **2005** | **3.5(2.28 - 5.12)** | **3.71(3.18 - 4.24)** | **7.23(6.8 - 7.66)** | **6.69(6.56 - 6.81)** | **5.07(4.62 - 5.51)** |
| **2006** | **4.22(2.94 - 5.88)** | **3.49(2.99 - 3.99)** | **6.77(6.36 - 7.18)** | **6.19(6.07 - 6.31)** | **4.45(4.04 - 4.86)** |
| **2007** | **4.65(3.24 - 6.47)** | **3.45(2.96 - 3.93)** | **6.66(6.26 - 7.07)** | **5.79(5.67 - 5.9)** | **3.95(3.58 - 4.32)** |
| **2008** | **3.45(2.3 - 4.99)** | **3.12(2.67 - 3.57)** | **6.59(6.19 - 6.98)** | **5.56(5.44 - 5.67)** | **3.74(3.39 - 4.09)** |
| **2009** | **2.59(1.63 - 3.93)** | **3.31(2.87 - 3.75)** | **5.74(5.38 - 6.1)** | **5.27(5.16 - 5.38)** | **3.37(3.04 - 3.69)** |
| **2010** | **4.36(3.04 - 6.06)** | **2.87(2.46 - 3.28)** | **5.61(5.25 - 5.96)** | **5.07(4.96 - 5.18)** | **3.67(3.34 - 4)** |
| **2011** | **3.52(2.42 - 4.94)** | **2.45(2.09 - 2.8)** | **5.11(4.77 - 5.44)** | **4.68(4.58 - 4.78)** | **3.01(2.72 - 3.29)** |
| **2012** | **3.19(2.18 - 4.5)** | **2.75(2.38 - 3.12)** | **4.79(4.47 - 5.1)** | **4.58(4.48 - 4.68)** | **2.95(2.67 - 3.22)** |
| **2013** | **2.97(2.05 - 4.17)** | **2.46(2.12 - 2.8)** | **4.33(4.03 - 4.62)** | **4.35(4.25 - 4.45)** | **2.43(2.19 - 2.67)** |
| **2014** | **3.32(2.32 - 4.6)** | **2.12(1.82 - 2.42)** | **4.37(4.08 - 4.66)** | **4.1(4.01 - 4.19)** | **2.77(2.52 - 3.02)** |
| **2015** | **4.06(2.99 - 5.38)** | **2.24(1.94 - 2.54)** | **4.03(3.75 - 4.3)** | **3.95(3.86 - 4.04)** | **2.58(2.35 - 2.82)** |
| **2016** | **3.28(2.37 - 4.44)** | **2.05(1.77 - 2.32)** | **3.94(3.68 - 4.21)** | **3.87(3.78 - 3.96)** | **2.44(2.22 - 2.67)** |
| **2017** | **3(2.12 - 4.11)** | **1.74(1.49 - 1.98)** | **3.91(3.65 - 4.16)** | **3.75(3.66 - 3.83)** | **2.29(2.08 - 2.5)** |
| **2018** | **2.87(2.04 - 3.92)** | **1.71(1.47 - 1.95)** | **3.72(3.47 - 3.97)** | **3.78(3.69 - 3.86)** | **2.05(1.86 - 2.25)** |
| **2019** | **2.1(1.45 - 2.95)** | **1.75(1.52 - 1.98)** | **3.66(3.42 - 3.91)** | **3.75(3.67 - 3.84)** | **2.27(2.07 - 2.47)** |
| **2020** | **3.28(2.44 - 4.31)** | **1.99(1.75 - 2.24)** | **4.26(4 - 4.52)** | **3.89(3.8 - 3.98)** | **2.29(2.1 - 2.49)** |
| **Average Age-Adjusted Rate** | **3.79(2.62 - 5.32)** | **3.15(2.70 - 3.59)** | **5.97(5.61 - 6.33)** | **5.52(5.41 - 5.63)** | **3.70(3.36 - 4.05)** |

# Supplemental Table 9:

Chronic Ischemic Heart Disease Among Neoplasms-Related Age-Adjusted Mortality Rates per 100,000 Stratified by State in the United States, 1999 to 2020

| **State** | **Age-Adjusted Rate (95% CI)** |
| --- | --- |
|  | **1999-2020** |
| **Alabama** | **3.03(2.9 - 3.15)** |
| **Alaska** | **3.49(2.99 - 4)** |
| **Arizona** | **3.5(3.39 - 3.62)** |
| **Arkansas** | **3.31(3.14 - 3.47)** |
| **California** | **6.64(6.57 - 6.72)** |
| **Colorado** | **3.49(3.35 - 3.64)** |
| **Connecticut** | **5.94(5.75 - 6.13)** |
| **Delaware** | **5.53(5.15 - 5.92)** |
| **District of Columbia** | **7.29(6.72 - 7.87)** |
| **Florida** | **4.42(4.35 - 4.48)** |
| **Georgia** | **2.87(2.77 - 2.97)** |
| **Hawaii** | **3.9(3.65 - 4.15)** |
| **Idaho** | **3.47(3.22 - 3.72)** |
| **Illinois** | **4.37(4.28 - 4.46)** |
| **Indiana** | **4.68(4.54 - 4.82)** |
| **Iowa** | **5.84(5.64 - 6.04)** |
| **Kansas** | **4.24(4.05 - 4.43)** |
| **Kentucky** | **3.95(3.8 - 4.1)** |
| **Louisiana** | **3.09(2.96 - 3.23)** |
| **Maine** | **4.76(4.49 - 5.03)** |
| **Maryland** | **6.21(6.04 - 6.38)** |
| **Massachusetts** | **4.15(4.03 - 4.27)** |
| **Michigan** | **5.57(5.46 - 5.69)** |
| **Minnesota** | **4.67(4.52 - 4.81)** |
| **Mississippi** | **4.37(4.17 - 4.57)** |
| **Missouri** | **4.36(4.23 - 4.49)** |
| **Montana** | **4.31(4 - 4.62)** |
| **Nebraska** | **5.51(5.24 - 5.77)** |
| **Nevada** | **3.75(3.55 - 3.96)** |
| **New Hampshire** | **5.08(4.78 - 5.39)** |
| **New Jersey** | **5.88(5.75 - 6)** |
| **New Mexico** | **3.81(3.59 - 4.04)** |
| **New York** | **9.59(9.48 - 9.7)** |
| **North Carolina** | **4.32(4.21 - 4.43)** |
| **North Dakota** | **5.99(5.55 - 6.42)** |
| **Ohio** | **6.27(6.16 - 6.39)** |
| **Oklahoma** | **6.54(6.33 - 6.75)** |
| **Oregon** | **4.22(4.06 - 4.38)** |
| **Pennsylvania** | **5.71(5.62 - 5.81)** |
| **Rhode Island** | **7.83(7.43 - 8.23)** |
| **South Carolina** | **3.47(3.33 - 3.61)** |
| **South Dakota** | **4.55(4.21 - 4.9)** |
| **Tennessee** | **4.83(4.69 - 4.97)** |
| **Texas** | **4.06(3.98 - 4.13)** |
| **Utah** | **2.34(2.16 - 2.51)** |
| **Vermont** | **7.09(6.59 - 7.6)** |
| **Virginia** | **3.51(3.4 - 3.62)** |
| **Washington** | **4.91(4.77 - 5.05)** |
| **West Virginia** | **7.01(6.73 - 7.3)** |
| **Wisconsin** | **4.32(4.19 - 4.45)** |
| **Wyoming** | **3.44(3.03 - 3.84)** |

# Supplemental Table 8:

Chronic Ischemic Heart Disease Among Neoplasms-Related Age-Adjusted Mortality Rates per 100,000 Stratified by Region in the United States, 1999 to 2020

| **Year** | **Age-Adjusted Rate (95% CI)** | | | |
| --- | --- | --- | --- | --- |
|  | **Northeast** | **Midwest** | **South** | **West** |
| **1999** | **11.2(10.86 - 11.53)** | **8.06(7.79 - 8.33)** | **6.64(6.43 - 6.84)** | **9.04(8.73 - 9.36)** |
| **2000** | **10.65(10.32 - 10.98)** | **7.6(7.34 - 7.86)** | **6.51(6.31 - 6.71)** | **8.46(8.15 - 8.76)** |
| **2001** | **9.97(9.66 - 10.29)** | **7.44(7.18 - 7.69)** | **6.18(5.99 - 6.38)** | **8.25(7.95 - 8.55)** |
| **2002** | **9.74(9.43 - 10.05)** | **7.01(6.77 - 7.26)** | **6.04(5.85 - 6.23)** | **8.02(7.73 - 8.31)** |
| **2003** | **9.37(9.07 - 9.67)** | **6.82(6.58 - 7.07)** | **5.82(5.63 - 6)** | **7.45(7.17 - 7.73)** |
| **2004** | **8.74(8.45 - 9.03)** | **6.56(6.32 - 6.79)** | **5.59(5.41 - 5.77)** | **7.05(6.79 - 7.32)** |
| **2005** | **8.52(8.23 - 8.8)** | **6.28(6.05 - 6.51)** | **5.28(5.1 - 5.45)** | **7.08(6.81 - 7.34)** |
| **2006** | **7.9(7.63 - 8.18)** | **6.03(5.8 - 6.25)** | **4.86(4.7 - 5.03)** | **6.37(6.13 - 6.62)** |
| **2007** | **7.67(7.41 - 7.94)** | **5.43(5.22 - 5.64)** | **4.59(4.44 - 4.75)** | **5.8(5.56 - 6.03)** |
| **2008** | **7.47(7.21 - 7.74)** | **5.44(5.23 - 5.65)** | **4.44(4.29 - 4.6)** | **5.4(5.18 - 5.62)** |
| **2009** | **6.87(6.62 - 7.12)** | **4.81(4.62 - 5.01)** | **4.28(4.13 - 4.43)** | **5.3(5.09 - 5.52)** |
| **2010** | **6.59(6.35 - 6.84)** | **4.75(4.56 - 4.94)** | **4.07(3.92 - 4.21)** | **5.11(4.9 - 5.32)** |
| **2011** | **5.98(5.75 - 6.21)** | **4.44(4.26 - 4.63)** | **3.69(3.56 - 3.83)** | **4.71(4.51 - 4.91)** |
| **2012** | **5.91(5.69 - 6.14)** | **4.33(4.14 - 4.51)** | **3.61(3.48 - 3.74)** | **4.56(4.37 - 4.75)** |
| **2013** | **5.37(5.16 - 5.59)** | **4.11(3.93 - 4.29)** | **3.38(3.25 - 3.5)** | **4.32(4.13 - 4.5)** |
| **2014** | **5.25(5.04 - 5.46)** | **3.8(3.63 - 3.97)** | **3.23(3.11 - 3.35)** | **4.19(4.01 - 4.37)** |
| **2015** | **4.9(4.7 - 5.11)** | **3.74(3.57 - 3.91)** | **3.17(3.05 - 3.29)** | **3.97(3.79 - 4.14)** |
| **2016** | **4.86(4.65 - 5.06)** | **3.57(3.41 - 3.73)** | **3.07(2.96 - 3.19)** | **3.85(3.68 - 4.01)** |
| **2017** | **4.47(4.28 - 4.66)** | **3.5(3.34 - 3.66)** | **3.11(3 - 3.23)** | **3.57(3.42 - 3.73)** |
| **2018** | **4.59(4.4 - 4.78)** | **3.54(3.38 - 3.7)** | **3.1(2.99 - 3.21)** | **3.32(3.17 - 3.47)** |
| **2019** | **4.43(4.24 - 4.62)** | **3.56(3.4 - 3.72)** | **3.17(3.06 - 3.28)** | **3.38(3.23 - 3.53)** |
| **2020** | **4.76(4.57 - 4.95)** | **3.5(3.34 - 3.65)** | **3.3(3.19 - 3.42)** | **3.58(3.42 - 3.73)** |
| **Average Age-Adjusted Rate** | **7.06(6.81 - 7.31)** | **5.27(5.07 - 5.47)** | **4.42(4.27 - 4.57)** | **5.58(5.36 - 5.80)** |

# Supplemental Table 7:

Chronic Ischemic Heart Disease Among Neoplasms-Related Age-Adjusted Mortality Rates per 100,000 Stratified by Urbanization in the United States, 1999 to 2020

| **Year** | **Age-Adjusted Rate (95% CI)** | |
| --- | --- | --- |
|  | **Urban** | **Rural** |
| **1999** | **8.68(8.53 - 8.83)** | **7.46(7.17 - 7.75)** |
| **2000** | **8.24(8.09 - 8.39)** | **7.27(6.98 - 7.55)** |
| **2001** | **7.93(7.79 - 8.08)** | **6.73(6.45 - 7)** |
| **2002** | **7.64(7.5 - 7.78)** | **6.71(6.44 - 6.98)** |
| **2003** | **7.26(7.12 - 7.4)** | **6.63(6.36 - 6.9)** |
| **2004** | **6.89(6.75 - 7.02)** | **6.4(6.13 - 6.66)** |
| **2005** | **6.71(6.58 - 6.84)** | **5.96(5.71 - 6.21)** |
| **2006** | **6.15(6.02 - 6.27)** | **5.82(5.57 - 6.07)** |
| **2007** | **5.75(5.63 - 5.86)** | **5.52(5.28 - 5.76)** |
| **2008** | **5.57(5.46 - 5.69)** | **5.12(4.89 - 5.35)** |
| **2009** | **5.23(5.12 - 5.34)** | **4.81(4.58 - 5.03)** |
| **2010** | **5.01(4.91 - 5.12)** | **4.85(4.63 - 5.07)** |
| **2011** | **4.54(4.45 - 4.64)** | **4.48(4.27 - 4.7)** |
| **2012** | **4.43(4.34 - 4.53)** | **4.41(4.21 - 4.62)** |
| **2013** | **4.14(4.04 - 4.23)** | **4.22(4.02 - 4.42)** |
| **2014** | **3.99(3.9 - 4.08)** | **3.98(3.78 - 4.17)** |
| **2015** | **3.78(3.7 - 3.87)** | **3.95(3.76 - 4.14)** |
| **2016** | **3.69(3.61 - 3.77)** | **3.75(3.56 - 3.94)** |
| **2017** | **3.53(3.45 - 3.61)** | **3.74(3.56 - 3.93)** |
| **2018** | **3.48(3.41 - 3.56)** | **3.68(3.49 - 3.86)** |
| **2019** | **3.49(3.41 - 3.57)** | **3.65(3.47 - 3.83)** |
| **2020** | **3.65(3.57 - 3.73)** | **3.95(3.76 - 4.13)** |
| **Average Age-Adjusted Rate** | **5.44(5.34 - 5.56)** | **5.14(4.91 - 5.37)** |

# Supplemental Table 4:

Chronic Ischemic Heart Disease Among Neoplasms-Related Age-Adjusted Mortality Rates per 100,000 Stratified by Place of Death in the United States, 1999 to 2020

| **Place of Death** | **Place of Death Code** | **Deaths** | **% Of Total Deaths** |
| --- | --- | --- | --- |
| **Medical Facility - Inpatient** | **1** | **67140** | **27.22%** |
| **Medical Facility - Outpatient or ER** | **2** | **22435** | **9.10%** |
| **Medical Facility - Dead on Arrival** | **3** | **2250** | **0.91%** |
| **Medical Facility - Status unknown** | **10** | **219** | **0.09%** |
| **Decedent's home** | **4** | **76659** | **31.08%** |
| **Hospice facility** | **5** | **5547** | **2.25%** |
| **Nursing home/long term care** | **6** | **62469** | **25.33%** |
| **Other** | **7** | **9455** | **3.83%** |
| **Place of death unknown** | **9** | **490** | **0.20%** |

|  | **Deaths** | | | **Crude Mortality Rate (95% Cl)** | | |
| --- | --- | --- | --- | --- | --- | --- |
| **Year** | **Young Adults** | **Middle Aged Adults** | **Older Adults** | **Young Adults** | **Middle Aged Adults** | **Older Adults** |
| 1999 | 31 | 998 | 13805 | 0.04 (0.05-0.03) | 1.65 (1.76-1.55) | 39.67 (40.33-39.01) |
| 2000 | 45 | 998 | 13290 | 0.05 (0.06-0.04) | 1.61 (1.71-1.51) | 37.98 (38.63-37.33) |
| 2001 | 33 | 981 | 12943 | 0.04 (0.05-0.03) | 1.52 (1.62-1.43) | 36.68 (37.31-36.04) |
| 2002 | 33 | 996 | 12665 | 0.04 (0.05-0.03) | 1.49 (1.71-1.51) | 35.65 (36.27-35.03) |
| 2003 | 47 | 1011 | 12270 | 0.06 (0.07-0.04) | 1.47 (1.62-1.43) | 34.21 (34.82-33.61) |
| 2004 | 46 | 1034 | 11764 | 0.06 (0.07-0.04) | 1.46 (1.59-1.4) | 32.49 (33.08-31.91) |
| 2005 | 44 | **1039** | 11604 | 0.05 (0.06-0.04) | 1.42 (1.56-1.38) | 31.66 (32.24-31.09) |
| 2006 | 33 | 993 | 10940 | 0.04 (0.04-0.02) | 1.32 (1.55--1.37) | 29.44 (29.99-28.86) |
| 2007 | 28 | 973 | 10439 | 0.03 (0.05-0.02) | 1.26 (1.51-1.33) | 27.6 (28.13-27.07) |
| 2008 | 21 | 998 | 10273 | 0.03 (0.04-0.02) | 1.27 (1.4-1.24) | 26.49 (27.0-25.98) |
| 2009 | 28 | 989 | 9767 | 0.03 (0.05-0.02) | 1.23 (1.34-1.18) | 24.65 (25.14-24.16) |
| 2010 | 28 | 1017 | 9560 | 0.03 (0.06-0.03) | 1.25 (1.35-1.19) | 23.74 (24.22-23.27) |
| 2011 | 23 | 963 | 8998 | 0.03 (0.04-0.02) | 1.16 (1.31-1.16) | 21.74 (22.19-21.29) |
| 2012 | 29 | 993 | 8968 | 0.04 (0.04-0.02) | 1.2 (1.32-1.17) | 20.79 (21.22-20.36) |
| 2013 | 34 | 933 | 8596 | 0.04 (0.05-0.03) | 1.12 (1.24-1.09) | 19.23 (19.64-18.82) |
| 2014 | 25 | 922 | 8445 | 0.03 (0.04-0,02) | 1.1 (1.27-1.12) | 18.26 (18.65-17.87) |
| 2015 | 23 | 932 | 8258 | 0.03 (0.04-0.02) | 1.11 (1.2-1.05) | 17.29 (17.66-16.92) |
| 2016 | 33 | 900 | 8209 | 0.04 (0.05-0.03) | 1.07 (1.17-1.03) | 16.67 (17.03-16.31) |
| 2017 | 22 | 919 | 8082 | 0.03(0.04-0.02) | 1.09 (1.18-1.04) | 15.89 (16.24-15.54) |
| 2018 | 22 | 898 | 8239 | 0.03 (0.04-0.02 | 1.07 (1.14-1) | 15.71 (16.05-15.37) |
| 2019 | 23 | 935 | 8443 | 0.03 (0.04-0.02 | 1.12 (1.19-1.05) | 15.62 (15.95-15.29) |
| 2020 | 27 | 965 | 9041 | 0.03 (0.04-0.02) | 1.17 (1.24-1.09) | 16.24 (16.58-15.91) |
| **Overall** | 678 | 21387 | 224599 | 0.04 (0.04-0.03) | 1.26 (1.28-1.25) | 24.19 (24.29-24.09) |

**Supplementary table 6: Chronic IHD Mortality, stratified by age group in adult cancer patients**

| **Year** | **CIHD among Common Solid Organ Cancers (Lung, Prostate, Breast, Colorectal)** | | **CIHD among Blood-Related Cancers (Leukemias, Lymphoma)** | |
| --- | --- | --- | --- | --- |
|  | **Total Deaths** | **Total Population** | **Total Deaths** | **Total Population** |
| 1999 | 8258 | 180408769 | 1727 | 180408769 |
| 2000 | 7893 | 181984640 | 1704 | 181984640 |
| 2001 | 7846 | 184305128 | 1599 | 184305128 |
| 2002 | 7676 | 186208028 | 1655 | 186208028 |
| 2003 | 7400 | 188090429 | 1642 | 188090429 |
| 2004 | 6944 | 190205384 | 1616 | 190205384 |
| 2005 | 6896 | 192551384 | 1626 | 192551384 |
| 2006 | 6514 | 195019359 | 1479 | 195019359 |
| 2007 | 6096 | 197403777 | 1460 | 197403777 |
| 2008 | 6084 | 199795090 | 1456 | 199795090 |
| 2009 | 5714 | 202107016 | 1390 | 202107016 |
| 2010 | 5516 | 203891983 | 1448 | 203891983 |
| 2011 | 5021 | 206592936 | 1427 | 206592936 |
| 2012 | 5010 | 208826037 | 1358 | 208826037 |
| 2013 | 4852 | 211085314 | 1298 | 211085314 |
| 2014 | 4625 | 213809280 | 1340 | 213809280 |
| 2015 | 4660 | 216553817 | 1261 | 216553817 |
| 2016 | 4442 | 218641417 | 1334 | 218641417 |
| 2017 | 4322 | 221447331 | 1291 | 221447331 |
| 2018 | 4342 | 223311190 | 1357 | 223311190 |
| 2019 | 4468 | 224981167 | 1398 | 224981167 |
| 2020 | 4794 | 226635013 | 1541 | 226635013 |

**Supplementary table 10:** Chronic IHD Mortality, stratified by cancer type in adult cancer patients

| **Solid Organ Cancer** | **AAMR (1999)** | **AAMR (2020)** | **Trends** |
| --- | --- | --- | --- |
| **Colorectal** | 1.1 | 0.2 | 1999-2003= APC (-4.81*), 2003-2016 = APC (-8.25*), 2016-2020 = APC (-3.16) |
| **Prostate** | 1.5 | 0.6 | 1999-2020= APC (-2.48), 2002-2018 = APC (-5.74*), 2018-2020 = APC (8.55*) |
| **Breast** | 0.9 | 0.3 | 1999-2017= APC (-6.02*), 2017-2020 = APC (-0.73) |
| **Lung** | 1.0 | 0.4 | 1999-2003 = APC (-2.87), 2003-2017= APC (-4.80*), 2017-2020= APC (0.43) |

**Supplementary table 11:** Detailed analysis of individual common solid organ cancers

| **Hematological cancers** | **AAMR (1999)** | **AAMR (2020)** | **Trends** |
| --- | --- | --- | --- |
| **Leukemia** | 0.09 | 0.05 | 1999-2020= APC (-3.97*) |
| **Lymphoma** | 0.33 | 0.14 | 1999-2020= APC (-4.53*) |

**Supplementary table 12:** Detailed analysis of hematological cancers
